# Balancing Strength and Ductility in Al Matrix Composites Reinforced by Few-Layered MoS_2_ through In-Situ Formation of Interfacial Al_12_Mo

**DOI:** 10.3390/ma14133561

**Published:** 2021-06-25

**Authors:** Lewen Fan, Lizhuang Yang, Dongdong Zhao, Liying Ma, Chunnian He, Fang He, Chunsheng Shi, Junwei Sha, Naiqin Zhao

**Affiliations:** 1 School of Materials Science and Engineering and Tianjin Key Laboratory of Composites and Functional Materials, Tianjin University, Tianjin 300350, China; fanlw96929@tju.edu.cn (L.F.); yanglz@tju.edu.cn (L.Y.); ddzhao@tju.edu.cn (D.Z.); lyma@tju.edu.cn (L.M.); cnhe08@tju.edu.cn (C.H.); fanghe@tju.edu.cn (F.H.); csshi@tju.edu.cn (C.S.); nqzhao@tju.edu.cn (N.Z.); 2 Collaborative Innovation Center of Chemical Science and Engineering, Tianjin 300350, China; 3 Key Laboratory of Advanced Ceramics and Machining Technology, Ministry of Education, Tianjin 300350, China

**Keywords:** aluminum, metal matrix composites (MMCs), MoS_2_ nanosheets, microstructure, mechanical property, strengthening mechanism

## Abstract

In this work, few-layered MoS_2_ (FLM) nanosheet-reinforced Al matrix composites are developed through powder metallurgy and hot extrusion. The microstructure, mechanical properties, and strengthening mechanisms have been systematically investigated. It is found that Al_12_Mo and Al_2_S_3_ can be formed in-situ during the sintering process, resulting in the improvement of interfacial bonding between FLM and Al matrix. With 1.5 wt.% of FLM addition, an improved tensile strength of 234 MPa with a high elongation of 17% can be obtained. Moreover, the strengthening mechanisms are also demonstrated to be grain refinement, dislocation strengthening, and load transfer, and the calculation indicates that load transfer is the main contribution factor. This work will inspire more new designs of metal matrix composites with balanced strength and ductility.

## 1. Introduction

Al matrix composites (AMCs) have attracted great attention in recent decades, and are widely used in fields including aircraft, automobile, and electronic packaging due to their high specific properties, fatigue performance, low thermal expansion coefficient, and wear coefficient [1,2,3]. In AMCs, nano-scale SiC [4], B4C [5], Al_2_O_3_ particles [6], and carbon nanomaterials including carbon nanotubes (CNTs) [7] and graphene (GN) [8,9] have been introduced into Al matrix to improve the mechanical and tribological properties of the composites. The above reinforcements can be categorized by dimension into zero-dimensional (0D) nanoparticles, one-dimensional (1D) nanomaterials, and two-dimensional (2D) nanosheets. Compared to 0D and 1D nanomaterials, 2D reinforcements with high specific surface area show promising prospects due to the ensured large contact area between the reinforcements and matrix, resulting in a more effective load transfer, which is the significant strengthening mechanism in AMCs [10]. As a typical 2D material, GN possesses unique mechanical properties, including high fracture strength of 130 GPa and high Young’s modulus (~1.0 TPa) [11]. With only 0.3 wt.% addition of GN nanosheets, 62% enhancement of tensile strength over the unreinforced Al matrix was achieved, showing great potential of GN for enhancing AMCs [12]. Thus, 2D GN is a promising reinforcement for enhancing AMCs. Besides, Shin [13] compared the strengthening behaviors of 2D GN with that of multi-walled carbon nanotubes (MWCNTs) and the results revealed that 2D GN with higher surface area was a much more effective reinforcement. The authors highlighted that the strength of the composites can be determined by the specific surface area of the reinforcements. That is to say, 2D nanomaterials with high specific surface area have natural advantages in reinforcing AMCs. Therefore, it is of great significance to explore more possibilities of 2D nano-reinforcement with high strengthening efficiency for reinforcing AMCs.

Recently, 2D transition metal chalcogenides (TMDs) including MoS_2_ and WS_2_ have attracted more attention, especially in fields including electronics, optoelectronics, and energy storage devices [14,15,16]. As a kind of typical TMDs, MoS_2_ possesses attractive merits, including high Young’s modulus of ~0.33 TPa [17] and breaking strength up to 23 GPa (monolayered MoS_2_) [18]. Besides, more and more work about facile preparation of MoS_2_ nanosheets has been reported through methods such as chemical vapor deposition (CVD) [19], ultrasonic exfoliation strategy [20], and chemical exfoliation with covalent modification [21]. The excellent mechanical properties and facile preparation methods of MoS_2_ nanosheets make them promising reinforcements for AMCs. Up to now, MoS_2_ nanosheets have been investigated as the reinforcements in polymer matrix composites, showing improvements in both strength and plasticity [22,23]. Peng et al. [22] fabricated MoS_2_ nanosheet-reinforced polyacrylonitrile (PAN) matrix composite fibers with significant improved mechanical properties. Only 2 wt.% of MoS_2_ nanosheets’ addition could lead to 70.4% and 54.2% improvements in tensile strength and fracture elongation for PAN fiber respectively, which provides us with a new strategy to solve the strength–toughness trade-off problem of metal matrix composites (MMCs). Huang et al. [24] prepared Al6061 matrix composites reinforced by using WS_2_, which possesses a similar structure as MoS_2_, showing a remarkable improvement in both tensile strength and elongation. It suggests that the MoS_2_ nanosheets may have great potential in strengthening and toughening MMCs. However, the true potential of MoS_2_ nanosheets in MMCs has not ever been investigated before. There are only a few studies reported in MMCs reinforced with bulk crystal MoS_2_ instead of nanosheets as solid lubricant for Cu, Fe, Al, and their alloys’ matrix [25,26,27,28,29,30,31]. With the addition of MoS_2_, the tribological properties were obviously improved with lower friction coefficient and wear rate, but the mechanical properties deteriorated due to the soft nature of bulk crystal MoS_2_. In the study of Cu-Sn alloy matrix composites reinforced by MoS_2_ [25], it was mentioned that the formation of brittle phase CuMoS3 increased the hardness and wear rate of the composites. Likewise, Furlan et al. [27] studied the reaction between iron-based matrices and the solid lubricant MoS_2_ during sintering and found that the friction coefficient of the sample containing unreacted MoS_2_ is higher than that with reacted MoS_2_. It can be concluded that the reaction between MoS_2_ and metal matrix resulted in the improved tribological and mechanical properties of the composites. Jaroslaw et al. [29] examined the hardness and tribological properties of Al6061-based composites with 2–15 vol.% MoS_2_ prepared through powder metallurgy. The results indicated that only the hardness of 2 vol.% MoS_2_/Al6061 was higher than that of unreinforced Al6061. The strengthening mechanisms were not deeply discussed. The mechanical and tribological properties of MoS_2_-AlSi10Mg self-lubricating composites were studied by Kannappan et al. The ultimate tensile strength decreased significantly due to various mechanisms initiated by MoS_2_, such as particle pull-out and crack propagation [32]. While, in the study of MoS_2_-Al2024 composites reported by Bhargavi, when the mass fraction of reinforcing particles in the matrix was increased to 4%, the hardness and tensile strength of the material increased. When the mass fraction of reinforcing particles in the matrix was increased to 5%, the hardness and tensile strength of the material decreased. Among the Al-MoS_2_ systems [29,32,33], the influence of MoS_2_ on the properties and mechanisms has not been studied in detail yet.

Similarly, the reported studies [28,29,30,31] of MoS_2_-reinforced AMCs focused on the influences of MoS_2_ on the tribological properties instead of the mechanical properties, and up to now the reaction between Al and MoS_2_ during the fabrication process is not clear. Therefore, it is necessary to investigate the effects of few-layered MoS_2_ nanosheets (FLM) on the microstructure and mechanical properties of Al matrix composites.

In this work, FLM-reinforced AMCs have been prepared by using powder metallurgy combined with the hot extrusion method. The microstructures and mechanical properties of the composites are systematically investigated, and the reaction between Al and MoS_2_ and the reinforcement effects of FLM on the composites are carefully studied. The tensile properties of AMCs are discussed based on different strengthening mechanisms.

## 2. Materials and Methods

### 2.1. Materials and Fabrication of FLM/Al Composites

**Figure 1:**
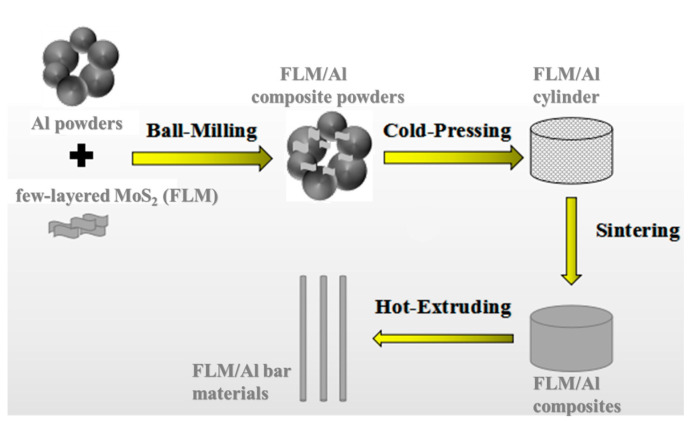
Schematic illustration of the fabrication process of FLM/Al bulk composites.

Al powders (purity > 99.7%, particle size: 9–11 µm), FLM nanosheets (diameter: 0.2–5 µm, layer: 1–10, thickness: 1–8 nm, Jiangsu XFNANO Materials Tech. Co., Ltd., Jiangsu, China), and commercial bulk crystal MoS_2_ (purity > 99.9 %, average diameter: ~3 µm) (XUXIN Chemical Industry Co., Ltd, Zhengzhou, China) were used in this work, as shown in Supplementary Figure S1. Figure 1 schematically presents the preparation process of the FLM/Al composites. Firstly, the FLM and pure Al powders with total mass of 12 g were placed in a 250 mL volume stainless-steel jar, 0.024 g of stearic acid (CH_3_(CH_2_)_16_CO_2_H) (Shanghai Aladdin Biochemical Technology Co., Ltd., Shanghai, China) was added as the process control agent (PCA), and 120 g of stainless-steel balls with a diameter of ~6 mm were taken as the ball-milled medium. Four kinds of FLM/Al powders by varying the weight fraction of FLM from 0.5 to 1.5 wt.% were prepared by the shift-speed ball-milling process at a speed of 200 rpm for 3 h, followed by 400 rpm for 2 h. The jars were filled with argon in the glovebox to avoid the oxidation of Al powders during the process. Secondly, the above Al-FLM composite powders were cold-compacted in a steel mold under a pressure of 600 MPa and maintained for 1 min at room temperature. Then, the cylinder with a diameter of 20 mm was sintered in a quartz tube furnace (Hefei KEJING Material Technology Co., Ltd., Hefei, China) in argon atmosphere at 630 or 550 °C for 1 h before being cooled inside the quartz tube to room temperature. After pre-heating at 570 °C for 30 min in the box-type furnace, the Al-FLM composites were hot-extruded to densify with an extrusion ratio of 16:1 to obtain Al-FLM bar materials. For comparison, pure Al samples were also produced with the same processing parameters. To verify the reaction products of MoS_2_ and Al, a bulk crystal MoS_2_-reinforced Al composite with a high MoS_2_ fraction of 10 wt.% was also fabricated under the same condition.

### 2.2. Characterization

The scanning electron microscopes (SEM, HITACHI S-4800 (Hitachi, Tokyo, Japan) and SU-1510 with tungsten filament) were used to observe the morphology of raw powders, ball-milled powders, and fracture surfaces of tensile test specimens. The Raman (in-Via microscope, Renishaw, London, England) test was conducted with a 532 nm laser, ranging from 500 to 3200 cm^−1^, which was used to detect MoS_2_ and its changes after the ball-milling process. X-ray diffraction (XRD, D8 Advance X-ray diffractometer (Bruker, Karlsruhe, Germany)) with Cu Kα radiation at a wavelength of 1.5406 Å was employed to identify the phase of the composite powders and bulk samples. The electron back-scattered diffraction (EBSD) technique integrated into FE-SEM (HITACHI S-7800) (Hitachi, Tokyo, Japan) was conducted to obtain the information of microstructure, especially grain size. A transmission electron microscope (TEM, JEOL JEM-2100F) (JEOL, Tokyo, Japan) equipped with scanning TEM (STEM) and an energy dispersive spectroscopy (EDS) detector was used to characterize the microstructure and interface of FLM/Al composites. Hardness tests (Vickers hardness) of bulk samples were conducted on an MH-6L test machine (EVERONE, Shanghai, China)) with a load of 200 gf and dwelling time of 5 s. Each sample was measured at 5 random points and standard deviation was calculated. The mechanical properties including strength and elongation were detected by uniaxial quasi-static tensile tests carried out with loading at a strain rate of 0.5 mm/min on a universal material testing machine (Lloyd (AMETEK) EZ 20) (AMETEK, Berwyn, PA, USA) at room temperature. A clip-on extensometer was used to measure the strain. The tensile specimens were machined to dog-bone-shaped with a gauge length of 15 mm and a gauge diameter of 3 mm.

## 3. Results

### 3.1. Microstructure of Composite Powders

**Figure 2:**
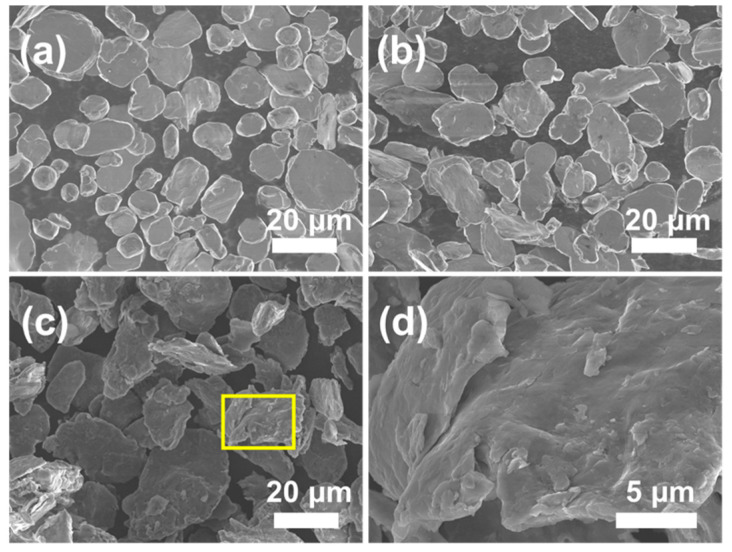
SEM images of ball-milled powders. (**a**) Pure Al, (**b**) 0.5 wt.% FLM/Al, (**c**) 1.5 wt.% FLM/Al, and (**d**) the amplification of the yellow box in (**c**).

**Figure 3:**
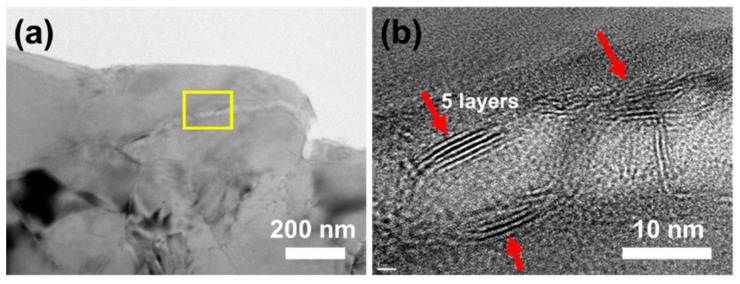
TEM images of 1.5 wt.% FLM/Al ball-milled powders: (**a**) FLM/Al composite powders, (**b**) enlarged view of yellow box in (**a**).

**Figure 4:**
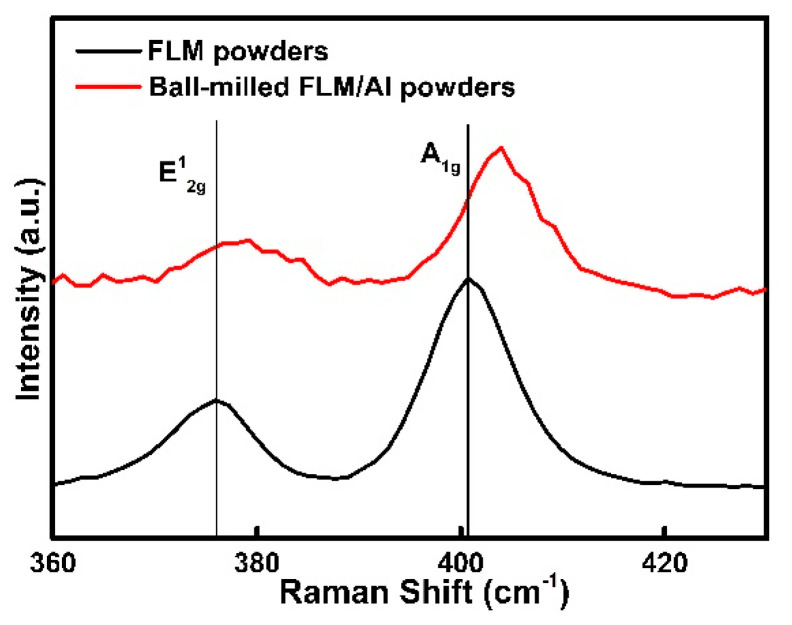
Raman spectra of raw MoS_2_ nanosheets and composite powders after the ball-milling process.

Figure 2 presents the morphology of pure Al and composite powders with different FLM contents after ball-milling. After the ball-milling process, both the pure Al and composites powders became flattened without obvious cold-welding. Compared to pure Al and 0.5 wt.% FLM-Al composite powders, the 1.5 wt.% FLM-Al composite powders shown in Figure 2c, d were subjected to more severe deformation, with larger diameter and much more fractured Al powders dispersed on the surface of Al flakes. The low strength and excellent deformability of pure Al powders causes them to suffer severe deformation without breakage, only flattening. With the introduction of FLM into the Al matrix, the plasticity of powders was reduced, making them break earlier than pure Al under the same process. It can be concluded that the ball-milling procedure was promoted by the addition of FLM. As shown in the TEM images of composite powders in Figure 3, MoS_2_ had several layers embedded into the Al matrix. The Raman spectra of raw FLM sheets and composite powders are displayed in Figure 4. The difference in intensity and position of the MoS_2_ characteristic peaks (E^1^_2g_ and A^1^_g_) between the two kinds of powders can be attributed to the influence of Al powders and the declined lateral size and thickness introduced by high shear forces during the ball-milling process [34]. Due to the high energy input during the ball-milling process for a long time, the solid reaction of Al and MoS_2_ powders will be induced [35]. However, in this study, it is difficult to identify the slight reaction because of the shorter ball-milling time and lower content of MoS_2_.

### 3.2. Mechanical Properties and Microstructure of Bulk Composites

#### 3.2.1. Mechanical Properties of Bulk Composites

**Figure 5:**
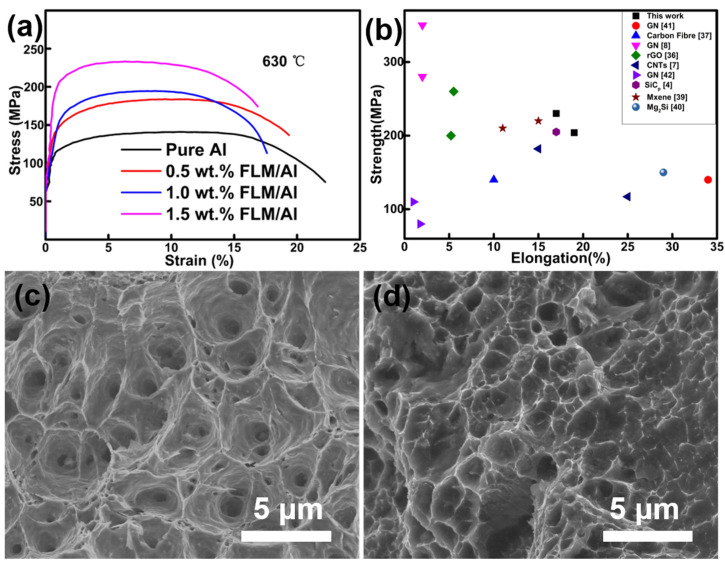
(**a**) Engineering stress–strain curves of bulk samples sintered at 630 °C, (**b**) comparison of tensile strength versus total elongation of composites of AMCs, (**c**,**d**) fracture surfaces of pure Al and 1.5 wt.% FLM/Al sintered at 630 °C.

Different contents of FLM-reinforced Al bulk composites were fabricated through the cold-compacted, sintering, and hot-extrusion processes, and then machined to tensile specimens. The engineering strain–stress curves are shown in Figure 5a. From the previous studies, the enhancement of strength tends to be accompanied by a great sacrifice of toughness [12,13]. It is noteworthy that the tensile strength of the composites improved along with the increase of FLM contents, with a slight decline of elongation. The 1.5 wt.% FLM/Al exhibited the highest ultimate tensile strength (UTS) of 234 MPa and yield strength (YS) of 210 MPa, showing great improvement compared to that of the unreinforced Al (UTS of 145 MPa and YS of 110 MPa). The elongation rate was 17%, which is at a relatively high level among the reported work [36,37,38]. The hardness of the bulk samples is shown in Supplementary Table S1. When the content of FLM was up to 1.5 wt.%, the hardness reached the maximum at 72 HV, which was similar to the result of the tensile strength trend. A summary of tensile properties’ data of composites based on the pure Al matrix in previous reports is shown in Figure 5b [4,7,8,36,37,38,39,40,41,42]. It can be seen that FLM/Al composites exhibited a good strength–ductility balance. Figure 5c,d show the fracture surface morphology of pure Al and 1.5 wt.% FLM/Al composites, respectively. Uniform dimple structures can be easily observed in the fracture surfaces, indicating a ductile fracture mode. Besides, the dimples of the composites are smaller and shallower than those of pure Al, indicating a higher strength and lower toughness than that of pure Al.

#### 3.2.2. Microstructure of Bulk Composites

**Figure 6:**
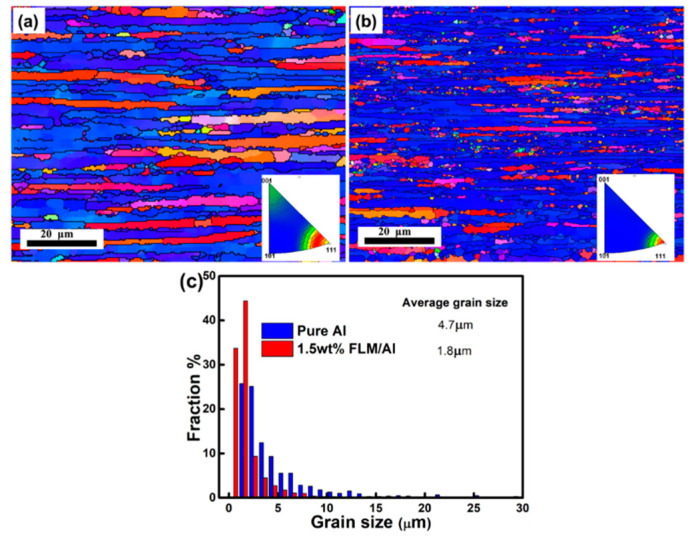
EBSD analysis results: IPF map of (**a**) pure Al, (**b**) 1.5 wt.% FLM/Al composite sintered at 630 °C, and (**c**) grain size distributions.

**Figure 7:**
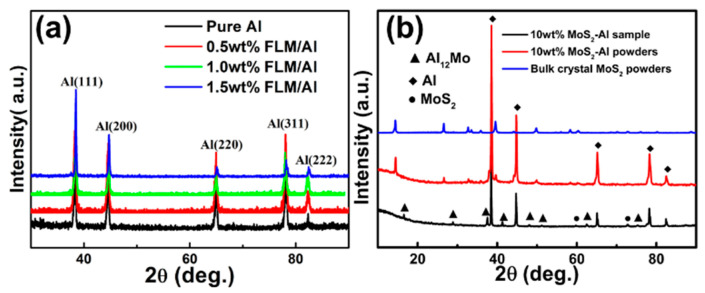
XRD analysis of (**a**) FLM/Al composites sintered at 630 °C, (**b**) bulk crystal MoS_2_ composite powders and bulk sample sintered at 630 °C.

In order to find out the reasons for the enhancement of the composites, the phase compositions and microstructures of bulk samples have been analyzed. Figure 6 displays the EBSD characterization results of pure Al and 1.5 wt.% FLM/Al composites after hot extrusion. It can be seen from the IPF maps in Figure 6a,b that owing to the hot extrusion, both pure Al and the composites exhibited textures with <111> orientated grains along the extrusion direction due to the grain rotations. However, the grain size of the sample has been significantly refined, as revealed by the grain size distribution in Figure 6c. The fine and near equiaxed grains from the diffraction contrast diagram and TEM image are shown in Supplementary Figure S2. There are two possible reasons for the grain refinement. The first one is the refinement introduced by the ball-milling process. As mentioned in Section 3.2, the FLM/Al composite powders were subjected to more severe deformation so that the initial finer grains can be obtained. The other reason is the effect of FLM on the nucleation and growth of Al grains. As for details, the FLM may stimulate the nucleation of recrystallization in the interface between FLM and Al during the hot-extrusion process due to the particle-stimulated nucleation mechanism, resulting in increased recrystallization. Meanwhile, the FLM would retard the migration of grain boundaries so that the growth of Al grains would be hindered. Similar results were also reported in SiC/Al [43] and GN/Al [9] composites. Figure 7 presents the XRD patterns of bulk samples with different FLM contents. The main peaks are corresponding to the Al, and no other diffraction peaks have been discovered including MoS_2_, resulted from the consumption of MoS_2_ and the low content of reaction products. To confirm the reaction of Al and MoS_2_, 10 wt.% bulk crystal MoS_2_-Al composites were fabricated under the same condition and tested by using XRD. The result is presented in Figure 7b. After the ball-milling process, the peaks of composite powders are corresponding to Al and MoS_2_ without any by-products. However, Takacs et al. investigated the ball-milling-induced reduction of MoS_2_ by Al according to the equation: Al + MoS_2_ = Al_2_S_3_ + Mo [35]. In this work, there are no peaks of Al_2_S_3_ and Mo in the XRD pattern of hybrid powders, while in the XRD pattern of 10 wt.% bulk crystal MoS_2_-Al sample, the main peak of MoS_2_ was absent and new peaks corresponding to Al_12_Mo appeared. According to the equilibrium phase diagrams [44] of Al-Mo and Al-S, when the weight fraction of Mo and S is under a certain value, the phase compositions are α-Al, Al_12_Mo, and Al_2_S_3_. Therefore, it is reasonable to conclude that the reaction products of Al and MoS_2_ in this work were Al_12_Mo and Al_2_S_3_.

**Figure 8:**
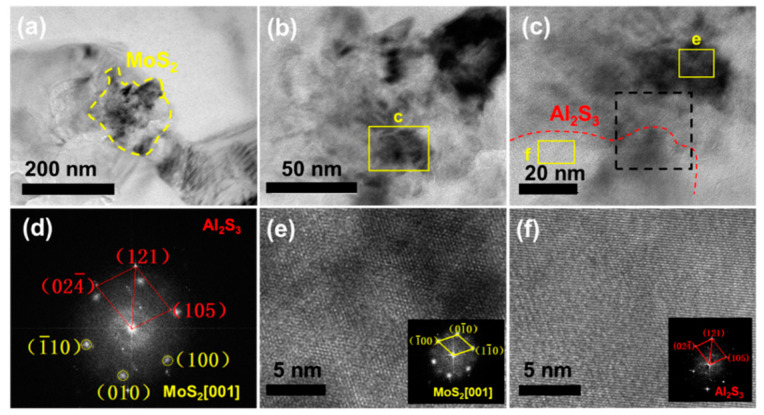
TEM microstructure of 1.5 wt.% FLM/Al composite sintered at 630 °C. (**a**,**b**) MoS_2_ in Al matrix, (**c**) HRTEM of yellow box in (**b**), (**d**) FFT of black box in (**c**), (**e**) HRTEM of MoS_2_, (**f**) HRTEM of Al_2_S_3._

**Figure 9:**
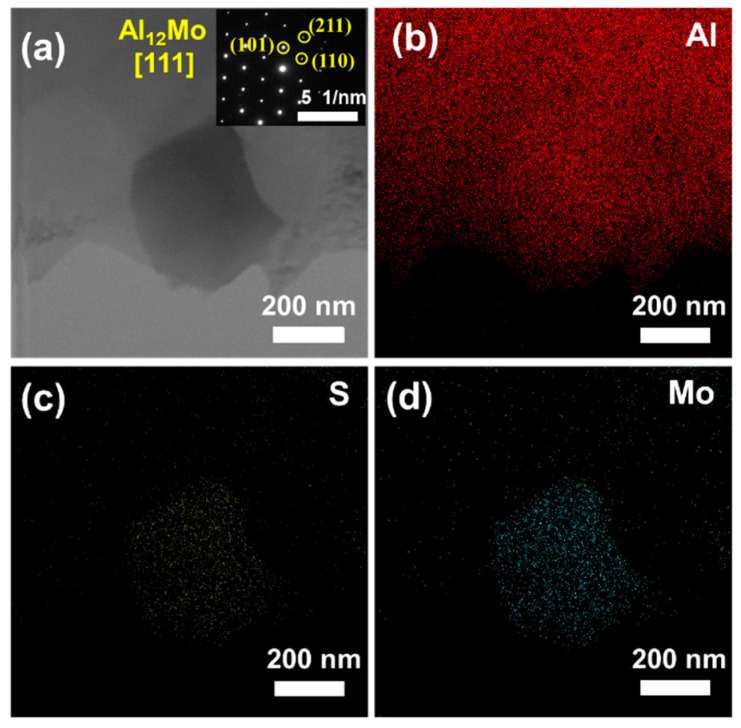
TEM images of the Al_12_Mo particles in 1.5 wt.% FLM/Al composite sintered at 630 °C. (**a**) Morphology of Al_12_Mo particles, and the inset is the SAED of part of (**a**), (**b**–**d**) element mapping of Al, S, and Mo.

Figure 8a,b present the TEM microstructure of the 1.5 wt.% FLM/Al composite. The MoS_2_ reinforcements preferred to locate at grain boundaries, as shown in Supplementary Figure S3. The formation of second phases at the grain boundaries were connected with grain boundary wetting transitions, thus affecting the properties of the material [45,46,47]. The HRTEM image of MoS_2_ is shown in Figure 8c and the fast Fourier transform (FFT) is shown in Figure 8d. The reaction products of Al_2_S_3_ around the FLM can promote the strong bonding between the Al matrix and FLM, which ensures that the load and strain can be effectively transferred. The lattice images of MoS_2_ and Al_2_S_3_ are shown in Figure 8e,f. Besides, we have found the Al_12_Mo particles in the Al matrix, as shown in Figure 9. The inset image in Figure 9a shows the selected area electron diffraction (SAED) of the Al_12_Mo particle. Al_12_Mo has an elastic modulus twice that of the Al matrix and high stability [48]. Recently, the in-situ synthesis of the intermetallic compound Al_12_Mo has been proven to be able to dramatically enhance mechanical properties of the Al matrix [38,49]. In the Al-ZrO_2_-MoSi_2_ system [49], the reaction of Al and MoSi_2_ to form Al_12_Mo was started at 728 °C. The in-situ-formed Al_12_Mo particles can act as the barrier for the dislocation movement even at the elevating temperature because of their thermal stability. In the present study, Al_12_Mo particles did not coarsen when constrained by the low temperature and short time. According to the EDS results (Figure 9b–d), it should be noted that the S element distributed at the same position as Al_12_Mo. The reaction controlled by the diffusion of atoms occurred at solid-solid state so that the low diffusion speed of atoms resulted in an incomplete reaction, which can account for the fact that the S element remains in Al_12_Mo phase. A similar result was detected in the 10 wt.% bulk crystal MoS_2_/Al composite, as shown in Supplementary Figure S2.

### 3.3. Enhancement by Interfacial Reaction Process

Based on the above analysis and discussions, the reaction products are Al_2_S_3_ and Al_12_Mo under the applied process parameters. The reaction progress can be expressed as the following Equations (1)–(3):

1.5 MoS_2_ (s) + 2Al(s) = 1.5Mo(s) + Al_2_S_3_ (s) ΔH = −231.5 kJ/mol (1)

12Al (s) + Mo (s) = Al_12_Mo (s) (2)

40 Al (s) + 3 MoS_2_ (s) = 3 Al_12_Mo (s) + 2 Al_2_S_3_ (s) (3)

Due to the low content of the reaction products, it is hard to identify the formation of Al_2_S_3_ and Mo in the XRD patterns of ball-milled powders, as shown in Figure 7b. However, Takacs et al. [35] have reported that the reaction (1) would be induced by ball-milling. Therefore, it is reasonable to conclude that during the ball-milling process, only Equation (1) was slightly carried out in this study. As revealed by the study of Li [50], MoS_2_ firstly reacted with Al and produced Al_2_S_3_ and Mo. Then, the Al combined with Mo to generate a series of aluminides with the rising temperature. According to the XRD results, the reaction product Al_12_Mo can be found so that the reaction can be expressed by Equation (2). Equation (3) can be obtained by the combination of Equations (1) and (2). However, there are no Al_2_S_3_ peaks in the XRD pattern of the 10 wt.% MoS_2_/Al bulk sample, since the amount of Al_2_S_3_ in the composites is too low to be detected, possibly. Assuming that the reduction of MoS_2_ is complete, the weight percentage of Al_2_S_3_ in the 10 wt.% MoS_2_/Al bulk sample is calculated to be 6.2%, according to Equation (3). Besides, the Al_2_S_3_ is very easy to hydrolyze. Therefore, the content of Al_2_S_3_ was far less than 6.2% with the uncomplete reaction and the decomposition of Al_2_S_3_. In the study of Li [50], similar processes have also been studied, where the mixed powders of MoS_2_ and Al were annealed at different temperatures. According to the research results, when the sample was annealed at 630 °C, Al_2_S_3_, Mo, and Mo-rich aluminides (Al_5_Mo and Al_12_Mo) were formed. However, different from the experiment results of Li [50], there was no formation of fragile Al_5_Mo phase and Mo in the present study owing to the excessive amount of Al. Besides, the three reactions are constrained by the diffusion of atoms in solid Al due to the temperature being lower than the melting point of Al.

**Figure 10:**
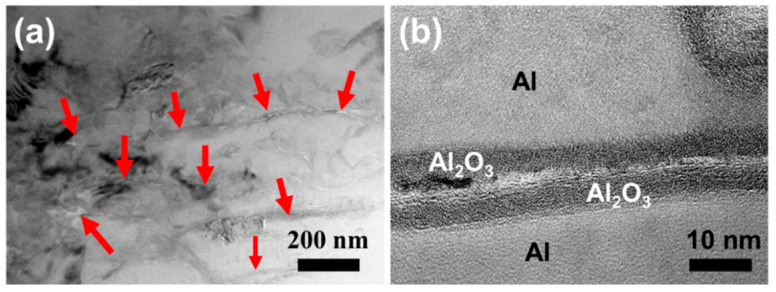
(**a**) TEM images of the interface structure in 1.5 wt.% FLM/Al composite at 550 °C, (**b**) HRTEM image of a FLM/Al interface.

In order to better understand the reaction process, we observed the microstructure of the 1.5 wt.% FLM/Al composite sintered at 550 °C. As shown in Figure 10a, the MoS_2_ nanosheets (see red arrows) with higher contrast in the bright-field TEM image are easily distinguished from the Al matrix and are likely to be parallel to each other. Figure 10b displays the morphology of an FLM/Al interface. An amorphous Al_2_O_3_ layer with a thickness of ~10 nm can be observed, indicating that the FLM nanosheets were surrounded by the thin Al_2_O_3_ layers when sintered at the low temperature. Under the condition of sintering at 550 °C, there were no obvious reaction products, suggesting that atomic diffusion was inhibited so that it was difficult for the reaction to occur. The weaker bonding in FLM/Al than that in in-situ-formed Al_12_Mo-Al and Al-Al_2_S_3_-FLM resulted in the interfacial debonding and less load transferring. It is consistent with the poor mechanical properties of the 1.5 wt.% FLM/Al composite at 550 °C (Supplementary Figure S3a). High-temperature sintering can break the inhibition of the oxide layer on the surface of Al powders and accelerate atomic diffusion so that the reaction can be promoted at a higher temperature of 630 °C (near the Al melting point). During the sintering process, the liquid Al may be momentarily present, and Al_2_O_3_ layers will partially fracture [51]. The MoS_2_ nanosheets will directly contact with Al, creating a favorable condition for the reaction. The reaction between MoS_2_ nanosheets and Al can enhance the interface bonding and the products can bear the load from the Al matrix. Given the lower content of Al_2_S_3_ and lower bulk modulus than that of Al_12_Mo (Al_2_S_3_: ~33 GPa; Al_12_Mo: ~91 GPa [52]), the enhancement was mainly attributed to the in-situ formation of Al_12_Mo. Both the TEM and XRD results confirmed the formation of Al_12_Mo. The strong chemical bonding provided Al_12_Mo particles with high hardness and modulus an advantage to take loads from the Al matrix. It can act as the barrier of dislocation movement during the tensile procedure. Meanwhile, the residual MoS_2_ sheets well-bonded with the Al matrix can also bear the load.

### 3.4. Strengthening Mechanisms Contribution Calculation

The enhancement of mechanical properties by the introduction of FLM can be attributed to several reasons. There is a collaboration of multiple strengthening mechanisms, including grain refinement, dislocation strengthening, Orowan looping mechanism, and load transfer, which have been documented in the previous work [53,54,55]. As displayed in Supplementary Figure S4, MoS_2_ distributed in the grain boundaries. Therefore, the contribution of the Orowan mechanism was not considered. The contribution of each mechanism was calculated to reveal the major reason for the reinforcement effects.

(i) Grain refinement

As mentioned above, the grains of the 1.5 wt.% FLM/Al composite have been significantly refined. Its contribution to strength (Δ*σ_GR_*) can be calculated by the Hall–Petch relationship, which is inversely proportional to the square root of the grain size [56] (Equation (4)):

(4) $$\Delta \sigma_{GR}=k\left(d_{com}^{-0.5}-d_{Al}^{-0.5}\right)$$

where *k* is the Hall–Petch coefficient (0.078 MPa*m^0.5^ for Al), and *d_com_* and *d_Al_* are the average grain sizes (4.7 μm for pure Al and 1.8 μm for 1.5 wt.% FLM/Al composite, respectively). The results of average grain sizes are shown in Figure 6c. The value of Δ*σ_GR_* is equal to 22 MPa.

(ii) Dislocation strengthening

During the deformation process, the movement of dislocations will be suppressed by the FLM with high specific surface area. As shown in Supplementary Figure S3b, the dislocation lines are blocked at the interface between the Al matrix and FLM. The strengthening effects caused by dislocation can be evaluated by the following Equation (5) [57]:

(5) $$\Delta \sigma_{dis}=\alpha \mathrm{MGb}\left(\rho_{com}^{0.5}-\rho_{Al}^{0.5}\right)$$

where *α* is the geometric constant and the value is 0.2 for F.C.C. metals, *M* is the orientation factor and the value is 3.06 for Al, *G* is shear modulus and is 26 GPa for Al, *b* is Burgers Vector and is 0.286 nm for Al, and *ρ_com_* and *ρ_Al_* are dislocation densities of the composites and Al after tensile deformation, respectively.

It is challenging to obtain dislocation density from TEM results, so the XRD tests were applied to the samples after tensile tests to directly measure the dislocation density by the Williamson–Hall (W-H) method [13,58,59]. The method is based on the principle that the increment of dislocation density is accompanied by the broadening of diffraction peaks. The expression of the W-H relationship is shown in Equation (6):

(6) $$B\cos\theta =\varepsilon \sin\theta +\frac{k\lambda }{d}$$

where *B* is the full width of the diffraction peak at half-maximum (FWHM) and *θ* is the Bragg angle, which can be easily obtained by the XRD patterns of samples. *K* is a constant of 0.9 and *λ* is the wavelength of Cu K_α_ radiation, 1.5418 Å. *d* is grain size and *ε* is micro-strain. The values of *d* and *ε* can be calculated by the slope and intercept of the fitting line of *B*cos*θ* vs. sin*θ* according to Equation (6). The calculated results are shown in Supplementary Table S2. The plot of fitting curves is displayed in Supplementary Figure S5. After the above operations, the dislocation density, *ρ*, can be obtained by the following Equation (7):

(7) $$\rho =\frac{2\sqrt{3}\varepsilon }{\mathrm{bd}}$$

The calculated values of dislocation density for pure Al and composite are equal to 4.16 × 10^14^ m^−2^ and 5.51 × 10^14^ m^−2^, respectively. Putting the values into Equation (5), the Δ*σ_dis_* for the 1.5 wt.% FLM-Al composite is equal to 14 MPa.

(iii) Load transfer

In general, the calculation of enhancement by the load transfer mechanism needs the results of the average aspect ratio (*S*) and the volume fraction (*V_f_*) of the reinforcement phase, as shown in the following Equation (8) [9]:

(8) $$\Delta \sigma_{LT}=\left(\frac{S}{4}-1\right)V_{f}\sigma_{m}$$

where *σ_m_* is the yield strength of the Al matrix. However, it is difficult to measure these values in the study. It is known that the enhancement contribution consists of three parts: (i) grain refinement, (ii) dislocation strengthening, and (iii) load transfer, which can be expressed by the following Equation (9):

(9) $$\Delta \sigma =\Delta_{GR}+\Delta_{\mathrm{dis}}+\Delta_{LT}$$

The enhancement by the load transfer mechanism can be estimated by the Equation (10):

(10) $$\Delta \sigma_{LT}=\Delta \sigma -\Delta_{GR}-\Delta_{\mathrm{dis}}$$

According to the above formulas, the values of Δ*σ_GR_*, Δ*σ_dis_*, and Δ*σ_LT_* of the 1.5 wt.% FLM-Al composite were calculated to be 22, 14, and 64 MPa, respectively. Apparently, the main contribution to strengthening is the load transfer mechanism, accounting for 64% of the total.

## 4. Conclusions

In this work, FLM-reinforced Al matrix composites with excellent mechanical properties have been successfully fabricated by the powder metallurgy process, and the influence of FLM addition on the microstructure and properties of AMCs was investigated. The following conclusions can be drawn from the results obtained:

(1) When the content of FLM in the composites reached 1.5 wt.%, the composites exhibited the highest ultimate tensile strength and yield strength of 234 and 210 MPa, respectively.
(2) It was found that MoS_2_ could react with Al to form Al_2_S_3_ and Al_12_Mo at 630 °C. The interface reaction generates strong chemical bonding between the matrix and reinforcement. The in-situ-formed Al_12_Mo played an important role in enhancement of Al matrix composites.
(3) There are three kinds of strengthening models: (i) grain refinement, (ii) dislocation strengthening, and (iii) load transfer. Load transfer is the dominant mechanism responsible for the improvement of strength based on the calculation of contribution by each mechanism.

## Data Availability

Not applicable.

## Supplementary Materials

The following are available online at https://www.mdpi.com/article/10.3390/ma14133561/s1, Figure S1: Morphology of the raw materials in the present work: (a) SEM image of raw Al powders, (b) SEM image of bulk crystal MoS_2_, (c,d) TEM image of FLM. Figure S2: (a) The diffraction contrast diagram of pure Al and (b) TEM image of composites. Figure S3: TEM image of FLM distributed in Al grain boundaries. Figure S4: EDS results of 10 wt.% bulk crystal MoS_2_-Al composite at 630 °C. Figure S5: (a) Tensile curve; (b) TEM image of 1.5 wt. %FLM/Al composite at 550 °C. Figure S6: Typical W-H plots of composite and pure Al sintered at 630 °C samples after tensile deformation. Table S1: Vickers Hardness of bulk samples sintered at 630 °C. Table S2: Calculated results of composite and pure Al samples after deformation by the W-H method.
