# Supplementary material for: Balancing Strength and Ductility in Al Matrix Composites Reinforced by Few-Layered MoS2 through In-Situ Formation of Interfacial Al12Mo
Source: Materials (Basel). 2021 Jun 25;14(13):3561. doi: 10.3390/ma14133561 (PMC8269559; doi:10.3390/ma14133561)
Supplement: Supplementary file 1 [file materials-14-03561-s001.zip › SM-materials-1260409-done.pdf]

Supporting Information-

Balancing strength and ductility in Al matrix composites reinforced by few-layered MoS_2 through in-situ formation of interfacial $Al_{12}Mo$

Lewen Fan [1], Lizhuang Yang [1], Dongdong Zhao [1], Liying Ma [1], Chunnian He [1,2,3], Fang He [1], Chunsheng Shi [1], Junwei Sha [1,*] and Naiqin Zhao [1,2,3]

[1] School of Materials Science and Engineering and Tianjin Key Laboratory of Composites and Functional Materials, Tianjin University, Tianjin 300350, China

[2] Collaborative Innovation Center of Chemical Science and Engineering, Tianjin 300350, China

[3] Key Laboratory of Advanced Ceramics and Machining Technology, Ministry of Education, Tianjin, 300350, China

* Correspondence: shajw@tju.edu.cn (J. Sha)

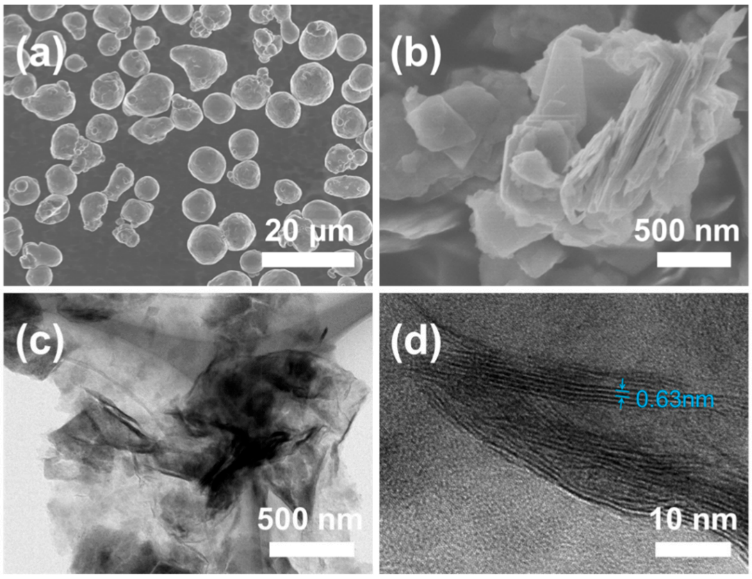

Figure S1. Morphology of the raw materials in the present work. (a) SEM image of raw Al powders; (b) SEM image of bulk crystal MoS_2; (c-d) TEM image of FLM

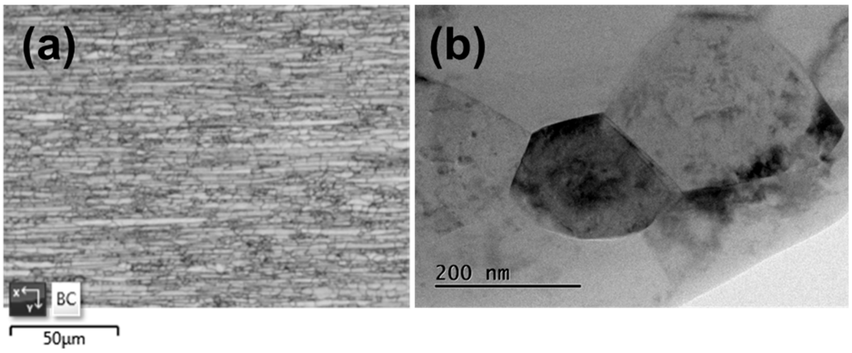

Figure S2. (a) The diffraction contrast diagram of pure Al and (b)TEM image of composites.

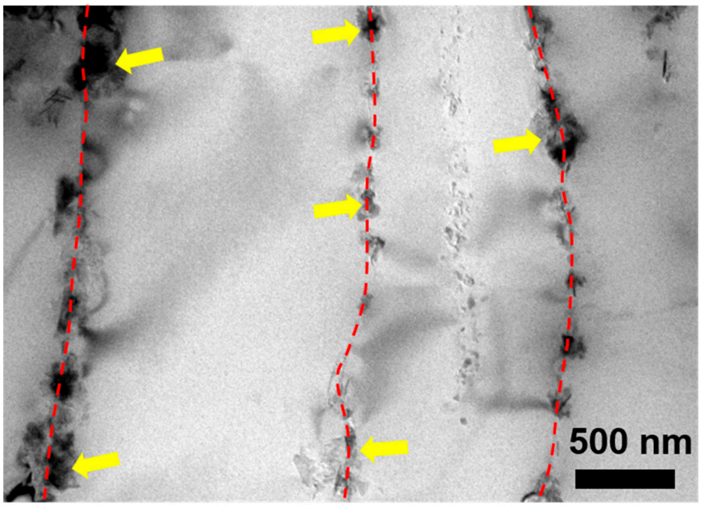

Fig. S3. TEM image of FLM distributed in Al grain boundaries

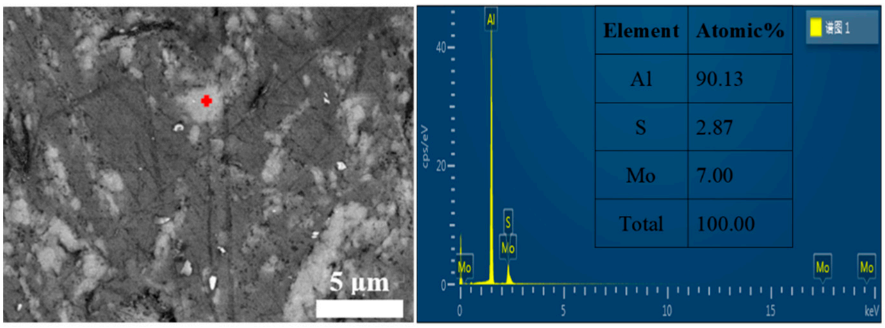

Figure S4. EDS results of 10 wt.% bulk crystal MoS_2-Al composite at 630 °C

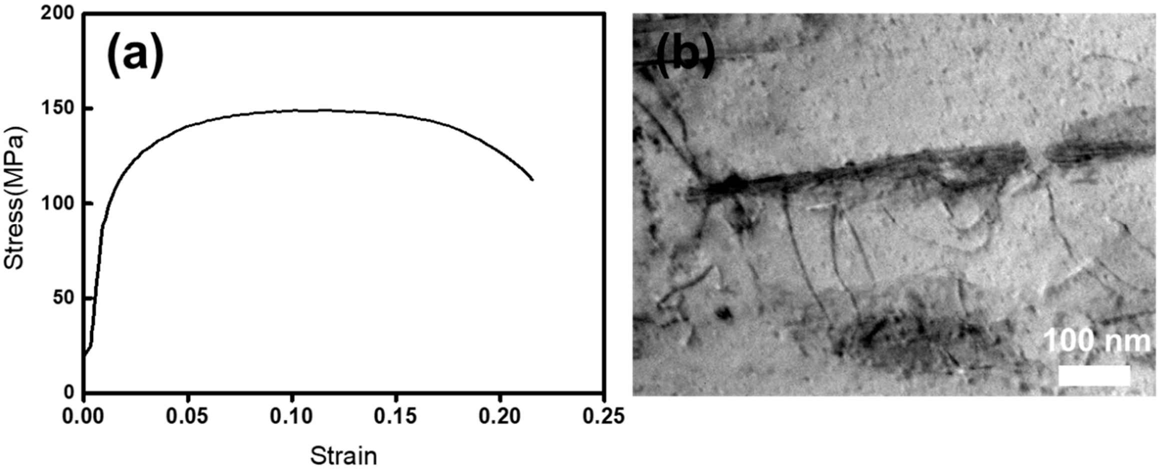

Figure S5. (a) Tensile curve; (b) TEM image of 1.5 wt. %FLM/Al composite at 550 °C

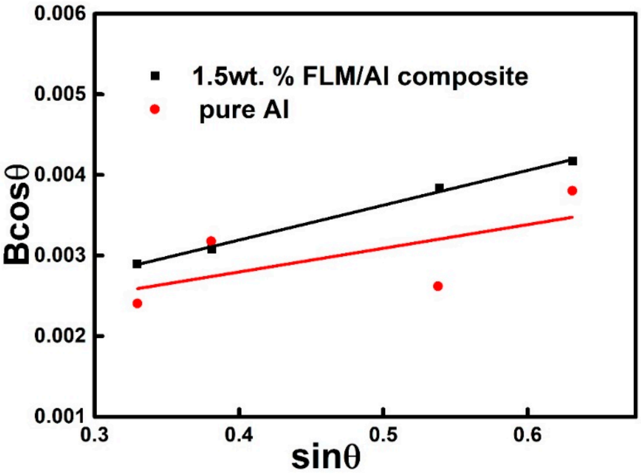

Figure S6. Typical W-H plots of composite and pure Al **sintered at 630 °C** samples after tensile deformation

Table S1 Vickers Hardness of bulk samples sintered at 630 °C

**Sample type**	Pure Al	0.5 wt. %FLM-Al	1.0 wt. %FLM-Al	1.5 wt. %FLM-Al
**HV**	46 ± 1.5	52 ± 0.5	57 ± 2.0	72 ± 1.5

Table S2. Calculated results of composite and pure Al samples after deformation by W-H method

**Sample type**	***ε*** **(slope)**	***kλ/d*** **(intercept)**	***ρ*** **(dislocation density, $10^{14}m^{-2}$)**
Pure Al	0.00294	0.00162	4.16
1.5 wt. %FLM-Al composite	0.00432	0.00146	5.51
